# Supplementary material for: Splice-Junction-Based Mapping of Alternative Isoforms in the Human Proteome
Source: Cell Rep. Author manuscript; Available in PMC 2020 Jan 15. (PMC6961840; doi:10.1016/j.celrep.2019.11.026)
Supplement: 2 [file NIHMS1546469-supplement-2.zip › DF1/PXD006675/RightAtrium/RightAtrium_3_MYBPC3_TDSHEDTGILDFSSLLK.pdf]

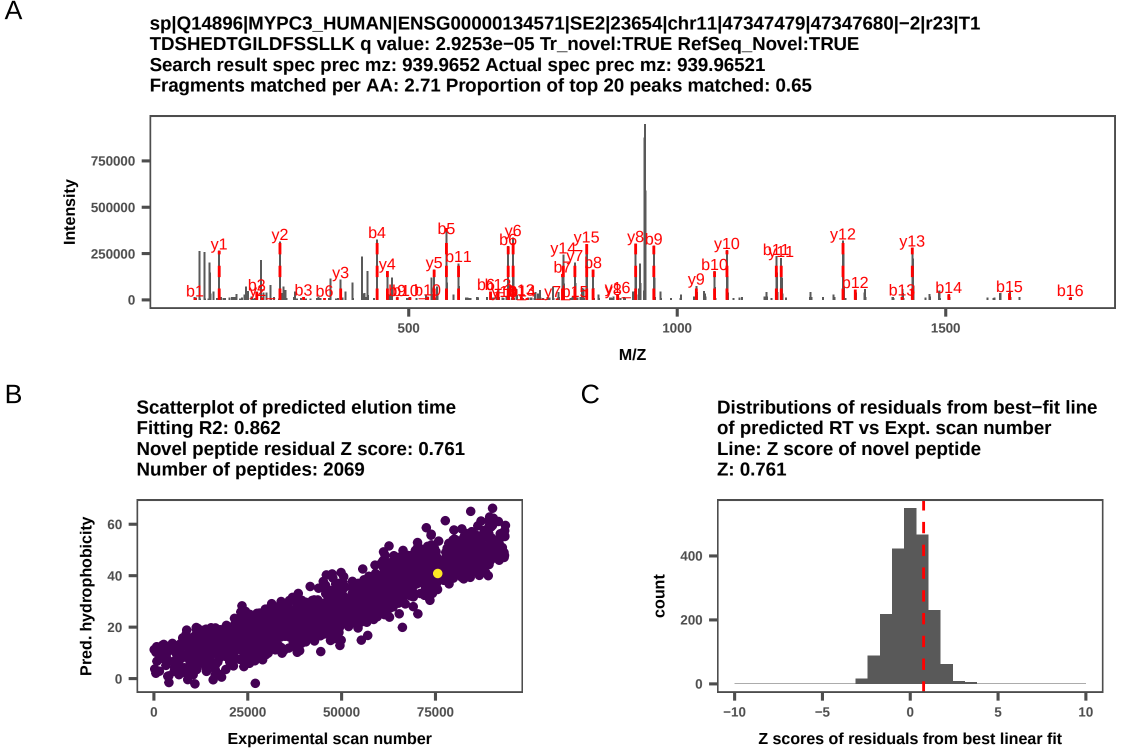
A
sp|Q14896|MYPC3_HUMAN|ENSG00000134571|SE2|23654|chr11|47347479|47347680|−2|r23|T1
TDSHEDTGILDFSSLLK q value: 2.9253e−05 Tr_novel:TRUE RefSeq_Novel:TRUE
Search result spec prec mz: 939.9652 Actual spec prec mz: 939.96521
Fragments matched per AA: 2.71 Proportion of top 20 peaks matched: 0.65
Intensity
750000
500000
250000
0
500
1000
1500
M/Z
y1
y2
b4
b5
y6
b6
y15
y14
y7
b7
b8
y8
b9
y10
b10
y9
b11
y11
y12
b12
y13
b13
b14
b15
b16
y3
y4
y5
b3
b1
B
Scatterplot of predicted elution time
Fitting R2: 0.862
Novel peptide residual Z score: 0.761
Number of peptides: 2069
Pred. hydrophobicity
60
40
20
0
0
25000
50000
75000
Experimental scan number
C
Distributions of residuals from best−fit line
of predicted RT vs Expt. scan number
Line: Z score of novel peptide
Z: 0.761
count
400
200
0
−10
−5
0
5
10
Z scores of residuals from best linear fit
